# Editorial: Insights in pediatric nephrology

**DOI:** 10.3389/fped.2025.1671358

**Published:** 2025-09-17

**Authors:** Michael L. Moritz, Agnieszka Swiatecka-Urban, Vera Hermina Koch

**Affiliations:** ^1^ Division of Pediatric Nephrology, Department of Pediatrics, Akron Children’s Hospital, Northeast Ohio Medical University, Akron, OH, United States; ^2^ Division of Pediatric Nephrology, Department of Pediatrics, University of Virginia School of Medicine, Charlottesville, VA, United States; ^3^ Pediatric Nephrology Unit, Department of Pediatrics, Instituto da Criança e do Adolescente do Hospital das Clínicas da Faculdade de Medicina da Universidade de São Paulo, Sau Paulo, Brazil

**Keywords:** pediatric nephrology, research, editorial, review, advances

**Editorial on the Research Topic**
Insights in pediatric nephrology

The field of pediatric nephrology continues to evolve, marked by both significant advancements and ongoing challenges. This collection of articles, assembled under the research topic “*Insights in Pediatric Nephrology*”, highlights emerging discoveries, innovative approaches, and evolving perspectives that are shaping the future of kidney care in children. Below is an overview of the thematic content of the collection, highlighting significant findings and emerging trends that are poised to shape the future of pediatric nephrology.

Two articles reported on the treatment of primary hyperoxaluria type 1 (PH1) with Lumasiran, an RNA interference (RNAi) therapeutic agent that reduces the hepatic production of oxalate by targeting glyoxylate metabolism (Frishberg et al., Taroni et al.). PH1 is a rare autosomal recessive inborn disorder in glyoxylate metabolism that is particularly devastating in young children as it results in kidney failure due to kidney stones and nephrocalcinosis which then leads to systemic oxalosis with the deposition of calcium oxalate crystals throughout the body. Frishberg et al. reported on the efficacy and safety of 30 months of Lumasiran treatment in 18 patients under 6 years of age enrolled in the phase 3 ILLUMINATE-B trial (Frishberg et al.). Lumasiran was found to be remarkably effective with a sustained reduction in urine oxalate of approximately 70% and demonstrated stable renal function, improvements in medullary nephrocalcinosis, and a low rate of kidney stone events. In addition, there were no serious adverse events related to Lumasiran use. Taroni et al. described a fascinating report on Lumasiran initiated at 10 days of age in a newborn prenatally diagnosed with PH1, as an older sibling also presented with PH1 at 2 months of age, and later required a liver and kidney transplant (Taroni et al.). Despite the early initiation of therapy, the child developed nephrocalcinosis and nephrolithiasis with elevated urine oxalate levels. It took approximately 9 months for urinary oxalate levels to normalize. At the 20-month follow-up, renal function and urine oxalate levels were normal, with improved nephrocalcinosis and no evidence of systemic oxalosis. These studies demonstrate the remarkable impact that biological drugs have had on rare pediatric diseases.

Two articles reported on the clinical utility of radiological imaging in assessing hemolytic uremic syndrome (HUS) and vesicoureteral reflux, respectively (Rink et al., Zhu et al.). Hemolytic uremic syndrome is an important cause of acute kidney injury in children, frequently requiring acute dialysis and sometimes resulting in CKD. It can be difficult to predict whether a child with HUS will have a severe clinical course requiring dialysis. Rink et al. retrospectively evaluated whether renal sonograms provide clinically meaningful information and predict the need for acute dialysis in 30 children with HUS (Rink et al.). The authors found that both increased kidney size and elevated resistive indices were predictive of the need for acute dialysis. Increased kidney size was the most predictive factor, with a kidney size >130% compared to mean values being associated with a 67% likelihood of needing dialysis, while a combination of a kidney size >130% and a resistive index >1.0 was associated with a 73% likelihood of needing dialysis. Interestingly, no children with a kidney size <130% required dialysis. These results suggest that renal sonography may be clinically useful for predicting the need for dialysis in children with HUS. Zhu et al. retrospectively evaluated renal damage using both Dimercaptosuccinic acid (DMSA) renal scintigraphy and Technetium-99m-Ethylenedicysteine (99mTc-EC) dynamic renal scintigraphy in 226 children diagnosed with primary vesicoureteral reflux (Zhu et al.). They demonstrated that scintigraphic abnormalities were common and that split renal function was much lower in kidneys that had reflux.

Two review articles were included, one discussing sodium glucose co-transporter 2 inhibitors and the other discussing renal abscesses in children (Portalatin et al., Sun et al.). Portalatin et al. discussed the growing interest in SGLT2 inhibitors as renoprotective agents in pediatric nephrology (Portalatin et al.). Originally developed for glycemic control in adults with type 2 diabetes, these agents have shown substantial renal and cardiovascular benefits in major chronic kidney disease (CKD) trials in adults, independent of glycemic status. Proposed mechanisms underlying the renal benefits include the restoration of tubulo-glomerular feedback, the reduction of intraglomerular pressure, the attenuation of inflammatory and fibrotic signaling, and the stabilization of podocyte and mesangial cell function. Although pediatric data remain limited, extrapolation from adult studies suggests a strong rationale for the use of SGLT2 inhibitors in select children and adolescents with proteinuric CKD and progressive glomerular diseases (e.g., IgA nephropathy, FSGS, and Alport syndrome). The authors advocated for disease-specific pediatric trials and provided interim recommendations for the cautious use of these agents in adolescents, emphasizing the importance of closely monitoring renal function and volume status. Sun et al. reviewed pediatric renal abscesses and reported on 12 cases (Sun et al.). Patients present with non-specific symptoms, such as fever and abdominal pain, and typically require an abdominal CT scan or magnetic resonance urography for diagnosis. Abscesses are typically less than 3 cm and respond to antibiotics, eliminating the need for surgery.

Finally, the European Society for Paediatric Nephrology (ESPN) conducted a multi-institutional cross-sectional analysis to evaluate the organization, workforce, and delivery of pediatric nephrology care in 48 European countries (Ehrich et al.). Despite progress in establishing specialized centers, transplant programs, and dialysis services, wide disparities persist. Over 50% of countries experience shortages of pediatric nephrologists, dialysis nurses, and clinical support staff. Common challenges include limited access to dialysis, inadequate transplant services for younger children, and a lack of multidisciplinary teams. ESPN calls for initiatives to standardize care, optimize training, and reduce inequities across regions. Their findings underscore the need for coordinated policies and investments to ensure equitable access to quality pediatric kidney care across Europe.

In conclusion, we congratulate the authors on their invaluable contributions to the field of pediatric nephrology. Their research and insights not only advance our understanding of complex kidney-related disorders in children but also highlight innovative therapeutic strategies and systemic improvements necessary to enhance patient care. Despite these significant strides, much work remains to be done to further advance the care of children with kidney disease. Continued research, collaboration, and investment are essential to addressing the existing challenges and disparities in pediatric nephrology, ensuring that all children have access to the highest quality of care. We look forward to future developments and the ongoing commitment of the pediatric nephrology community to improving outcomes for young patients worldwide.

